# Microvascular Impairment in Patients With Cerebral Small Vessel Disease Assessed With Arterial Spin Labeling Magnetic Resonance Imaging: A Pilot Study

**DOI:** 10.3389/fnagi.2022.871612

**Published:** 2022-05-19

**Authors:** Katja Neumann, Matthias Günther, Emrah Düzel, Stefanie Schreiber

**Affiliations:** ^1^German Center for Neurodegenerative Diseases (DZNE), Magdeburg, Germany; ^2^Fraunhofer Institute for Digital Medicine MEVIS, Bremen, Germany; ^3^MR-Imaging and Spectroscopy, University of Bremen, Bremen, Germany; ^4^mediri GmbH, Heidelberg, Germany; ^5^Institute of Cognitive Neurology and Dementia Research, Otto-von-Guericke University Magdeburg, Magdeburg, Germany; ^6^Institute of Cognitive Neuroscience, University College London, London, United Kingdom; ^7^Center for Behavioral Brain Science, Magdeburg, Germany; ^8^Department of Neurology, Otto-von-Guericke University Magdeburg, Magdeburg, Germany

**Keywords:** cerebral small vessel disease, perfusion, cerebral blood flow, arterial transit time, clearance, arterial spin labeling, magnetic resonance imaging, T1-relaxation

## Abstract

In this pilot study, we investigated microvascular impairment in patients with cerebral small vessel disease (CSVD) using non-invasive arterial spin labeling (ASL) magnetic resonance imaging (MRI). This method enabled us to measure the perfusion parameters, cerebral blood flow (CBF), and arterial transit time (ATT), and the effective T1-relaxation time (T1eff) to research a novel approach of assessing perivascular clearance. CSVD severity was characterized using the Standards for Reporting Vascular Changes on Neuroimaging (STRIVE) and included a rating of white matter hyperintensities (WMHs), lacunes, enlarged perivascular spaces (EPVSs), and cerebral microbleeds (CMBs). Here, we found that CBF decreases and ATT increases with increasing CSVD severity in patients, most prominent for a white matter (WM) region-of-interest, whereas this relation was almost equally driven by WMHs, lacunes, EPVSs, and CMBs. Additionally, we observed a longer mean T1eff of gray matter and WM in patients with CSVD compared to elderly controls, providing an indication of impaired clearance in patients. Mainly T1eff of WM was associated with CSVD burden, whereas lobar lacunes and CMBs contributed primary to this relation compared to EPVSs of the centrum semiovale. Our results complement previous findings of CSVD-related hypoperfusion by the observation of retarded arterial blood arrival times in brain tissue and by an increased T1eff as potential indication of impaired clearance rates using ASL.

## Introduction

In cerebral small vessel disease (CSVD), the physiology of small arteries (arterioles), capillaries, and venules is pathologically affected (Pantoni and Garcia, 1997; Wardlaw et al., 2013). Main subtypes of CSVD comprise hypertensive arteriopathy (HA) and cerebral amyloid angiopathy (CAA). Major risk factors for its sporadic forms are arterial hypertension and aging (de Leeuw et al., 1999; Dijk et al., 2008). CSVD is a pivotal cause for all-cause dementia and vascular contributions to cognitive decline (Zlokovic et al., 2020). *In vivo*, CSVD is diagnosed through magnetic resonance imaging (MRI) applying the Standards for Reporting Vascular Changes on Neuroimaging (STRIVE; Wardlaw et al., 2013). Increased frequencies of enlarged perivascular spaces (EPVSs) in the basal ganglia (BG), hippocampus, and white matter (WM; centrum semiovale, CSO) have been suggested as a common CSVD feature, probably indicating more early disease stages (Wardlaw et al., 2020; Gertje et al., 2021).

Perivascular spaces (PVSs) surrounding the perforating vessels are part of the brain's outflow routes, in which fluid transport takes place through arterial pulsation to remove metabolic by-products and proteins aiming to maintain tissue homeostasis (Rasmussen et al., 2021). Any dysfunction of that perivascular clearance has been linked to the aggregation of potentially toxic substances (Mucke and Selkoe, 2012), which has drawn the attention to drainage failure along the perforating vessels as a common mechanism underlying several neurovascular, neurodegenerative, or neuroimmunological disorders (Rasmussen et al., 2021).

It is possible to assess (perivascular) clearance function in humans by applying MRI, but thus far several suitable approaches demand the *invasive* intravenous or even intrathecal application of gadolinium compounds (Eide and Ringstad, 2015; Taoka and Naganawa, 2020). To facilitate larger cohort studies aiming to depict and understand clearance *in vivo* in older, and thus often much more vulnerable populations, there is an imperative need for new time-efficient and noninvasive approaches. Recently, Joseph et al. demonstrated the usage of an arterial spin labeling (ASL) MRI technique to determine impaired clearance rates in patients with Alzheimer's disease (AD; Joseph et al., 2020). ASL is a noninvasive imaging method, that uses magnetically labeled blood water as an endogenous tracer instead of exogenous contrast-agents (Williams et al., 1992). Images with a prior labeling of arterial blood (so-called tag images) are subtracted from images without labeling (control images). The resultant difference image is perfusion-weighted as signal contributions from tissue cancel out due to image subtraction. In pulsed ASL (PASL), blood water spins of a typically large slab outside the imaging slab are inverted simultaneously by a single radiofrequency (RF) pulse (Wong et al., 1998). This procedure generates a magnetically labeled blood bolus, which reaches regions (voxels) in the imaging slab after a certain time, called arterial transit time (ATT). Using multiple inversion times (TIs) to readout the signal, the inflow and outflow of the bolus can be sampled, whereas the curve is not only related to the shape of the bolus but also to the decay of signal after inversion.

In the study published by Joseph et al., the ASL signal decay over time was fitted linearly as an approximation of the exponential decay described by the Bloch equations (Bloch, 1946; Joseph et al., 2020). In this case, the referred ASL signal decay, called longitudinal or T1-relaxation, reflects interactions of labeled blood water protons with surrounding molecules. If the metabolic clearance is impaired, fluid from the (E)PVSs will retard the decay of the ASL signal, resulting in an increased effective longitudinal relaxation time (T1eff) compared to unimpaired clearance functions (Joseph et al., 2020).

There is overall growing evidence, that CSVD can be considered as a model disease of perivascular clearance failure, an assumption that is also based on the frequent detection of PVS enlargement in different regions of the microvascular diseased brain (Carare et al., 2020). To the best of our knowledge, there are, however, so far no studies applying ASL in patients with CSVD to determine regional drainage function at all and relate it to local perfusion and EPVS frequency in particular.

We here thus present a pilot study in patients with CSVD, showing, that noninvasive ASL MRI has the potential to assess impaired tissue clearance in patients with small vessel disease by fitting the ASL signal decay rate over time.

## Materials and Methods

### Participants

In total, 14 elderly participants (10 patients with CSVD and four controls) participated in this study approved by the Ethics Committee of the Otto-von-Guericke University Magdeburg, Germany (93/17). Signed informed consent was provided by each participant. CSVD diagnosis was based on the existence of cerebral microbleeds (CMBs), either in lobar or in deep and mixed regions, detected on prior 1.5 Tesla (T) iron/blood-sensitive MRI conducted for diagnostic work-up (Mugler and Brookeman, 1990; Linn et al., 2010). CAA was diagnosed according to the modified Boston criteria (Linn et al., 2010), and HA was evident in case of either deep or mixed, i.e., deep and lobar CMBs. Patients with CSVD had no neurological symptoms at all [modified Rankin Scale (mRS) 0] or no significant disability despite slight symptoms, and were able to carry out all usual duties and activities (mRS 1). The CSVD cohort included five cognitively normal subjects [mini mental state examination (MMSE) > 26], four of the patients fulfilled the criteria for mild cognitive impairment (MCI; 18 < MMSE ≤ 26), and in one patient MMSE data were missing (Supplementary Table 1). None of the patients suffered from large-artery stroke or any pathology of the large brain-supplying arteries. Controls were age-, sex-, and education-matched community dwelling elderly without CMBs and were cognitively normal, with one subject having missing MMSE scores (Table 1, Supplementary Table 1). Participants did neither have a history of a psychiatric disease, of a genetic neurological disease, nor of alcohol or drug abuse, and those with contraindications for MR measurements at 3-T field strength were excluded from this study.

**Table 1:** Demographical information of the study groups.

**Group**	**Controls** ***n* = 4**	**CSVD** ***n* = 10**
Females, *n* (%)	2 (50%)	4 (40%)
Median age (P25, P75) in [years]	71.0 (68.3, 76.8)	70.0 (62.5, 77.0)
Median education (P25, P75) in [years]	15.5 (13.5, 16.0)	13.0 (11.8, 13.8)
Median MMSE-score^*^ (P25, P75)	30.0 (30.0, 30.0)	27 (25, 28)
Median CSVD-score (P25, P75)	8.0 (7.9, 9.5)	23.4 (16.3, 28.7)
Hypertension yes/ no	2/ 2	10/ 0
Median systolic blood pressure (P25, P75) in [mmHg]	131.5 (130.0, 151.8)	130.0 (120.0, 157.5)
Median diastolic blood pressure (P25, P75) in [mmHg]	80.0 (77.0, 89.8)	80.0 (78.0, 80.5)

* *For one participant of the control group and for one participant of the CSVD group MMSE-score was missing*.

### Magnetic Resonance Imaging

All participants underwent an MRI acquisition at a 3-T Siemens MAGNETOM Skyra MRI Scanner (Siemens, Erlangen, Germany) with a 32-channel head coil (Nova Medical, Wilmington, Massachusetts, USA). The MRI protocol included a structural whole-brain T1-weighted MPRAGE sequence (Mugler and Brookeman, 1990) with 1 mm3 isotropic resolution, repetition time (TR) = 2,500 ms, echo time (TE) = 3.47 ms, and TI = 1,100 ms.

Additionally, a multi-inversion time (mTI) PASL scan with FAIR labeling, 3D-GRASE (Günther et al., 2005) readout and a suppression of background signal contributions with Q2TIPS to limit the bolus duration was obtained for every participant. Further imaging parameters of the PASL scan were: TR/TE = 3,800/22.32 ms, 16 slices with 5.5 mm thickness and 4 × 4 mm^2^ in-plane resolution, 256 × 192 mm^2^ field-of-view, band width = 2,298 Hz/Px, EPI factor 32, turbo factor = 12, two segments, two averaged measurements of 13 different TIs starting from 300 ms with an increment of 250 ms, and bolus length = 1,400 ms. The PASL scan included 5 M0-scans with TR = 4,360 ms and opposing phase encoding directions, respectively, for a distortion correction and cerebral blood flow (CBF) quantification. The acquisition time of the PASL scan was 7:08 min.

Cerebral small vessel disease markers according to STRIVE were evaluated visually based on a fluid-attenuated inversion recovery (FLAIR) sequence with 1 mm3 isotropic resolution, a T2-weighted TSE sequence with 0.5 × 0.5 mm^2^ in-plane resolution and 2 mm slice thickness, and a susceptibility weighted imaging (SWI) sequence with 0.6 × 0.6 × 2 mm3 voxel size (see “Visual MRI Analysis According to STRIVE” section).

All MRI measurements were conducted before noon to minimize perfusion signal variations caused by diurnal fluctuations (Ssali et al., 2016).

### Structural Pre-processing and Region-of-Interest Definition

Individual structural T1-weighted scans were pre-processed using tools of the Oxford Centre for functional MRI of the BRAIN (FMRIB) Software Library (FSL; Jenkinson et al., 2012). A tissue-type segmentation was done with the FAST tool (Zhang et al., 2001). Resulting tissue-type density maps were thresholded with a value of 0.9 and binarized to obtain gray (GM) and white matter (WM) masks of each participant. A subcortical structure segmentation [with FIRST tool (Patenaude et al., 2011)] was used to define additional regions-of-interest (ROIs) for the left (HCl) and right hippocampus (HCr) and for the BG. Structural segmentation results were also used for a partial-volume correction (Chappell et al., 2011) of the perfusion estimates.

### Arterial Spin Labeling Perfusion Analysis

Arterial spin labeling control and tag images, and related reference scans (M0 scans), were registered (Jenkinson et al., 2002) with FSL's FLIRT tool. A correction of a susceptibility-induced distortion was done with the help of related M0 scans with opposing phase encoding directions using FSL's TOPUP tool (Smith et al., 2004). For the estimation of quantitative perfusion parameters, CBF, and ATT, the BASIL toolkit was used (Chappell et al., 2009, 2010; Groves et al., 2009), which is based on the general kinetic model (Buxton et al., 1998) and a variational Bayes approach. This step included a partial volume correction based on tissue-type density maps calculated from individual structural data (Chappell et al., 2011). Proton density weighted M0 scans constitute the equilibrium magnetization and were used to transform estimated perfusion values from arbitrary to quantified physiological values [(ml/100 g/min) for CBF, and (s) for ATT].

### Estimation of the Effective T1-Relaxation Rates to Assess Perivascular Clearance

Arterial spin labeling MR images were used to evaluate a novel approach of assessing differences in the clearance rates between patients with CSVD and healthy elderly controls.

Therefore, individual ASL control and tag images were subtracted to obtain perfusion-weighted difference images for every TI. Only the ASL difference signal of the last three TIs (TI = 2,800/3,050/3,300 ms) was considered for this analysis, to reduce the impact of the bolus decay, or rather the arterial input function as much as possible. Mean signal intensities of the difference images of those TIs were calculated using individual masks for the BG, and for GM and WM.

The signal decay of the ASL difference signal over time for the last three TIs (**Figure 3**) was fitted exponentially using MATLAB R2017b (MathWorks, Sherborn, MA, USA). In cases with impaired clearance rates, a larger effective exponential coefficient (T1eff) is expected compared to unimpaired cases, as the exponential coefficient of the decay reflects the signal relaxation rate to thermal equilibrium, which differs between blood, tissue, and cerebrospinal fluid (CSF; see Macintosh and Graham, 2013; Bojorquez et al., 2017 for estimated values of T1).

### Visual MRI Analysis According to STRIVE

An MRI analysis of all subjects was performed in a semiquantitative manner according to STRIVE by a trained investigator (SS), blinded to all demographics and clinical information (Wardlaw et al., 2013). The images were evaluated using specific software (Mango for Dicom images; https://ric.uthscsa.edu/mango/) and established methods and scales (see below). With the exception of EPVS (see below), per participant, all available MRI slices were analyzed, respectively.

In short, CMBs were defined as small (2–10 mm), round, or oval hypointense lesions visible on SWI, categorized into lobar (frontal, temporal, parietal, occipital, and insula), deep (BG, thalamus, internal capsule, external capsule, corpus callosum, deep and periventricular WM), and infratentorial (cerebellum and brainstem) applying the Microbleed Anatomical Rating Scale (MARS; Gregoire et al., 2009). Intracerebral hemorrhages (ICHs; >10 mm) was classified as lobar or deep according to the Cerebral Hemorrhage Anatomical RaTing inStrument (CHARTS; Charidimou et al., 2017a), visible on SWI. Subarachnoid hemorrhages (SAHs) and cortical superficial siderosis (cSS) were defined as SWI hypointensities either in the subarachnoid space or in the superficial cortical layers. SAH/cSS was scored per hemisphere according to the cSS multifocality scale as absent (0), focal (1), or multifocal/disseminated (2), and the sum score of both hemispheres is given (Charidimou et al., 2017b). WM hyperintensities (WMHs) of vascular origin were graded in FLAIR images applying the FAZEKAS scale (WMH_Faz), considering the deep WM and periventricular WM separately (0 = absent, 1 = punctuate foci/pencil-thin lining, 2 = beginning confluence/smooth halo, 3 = large confluent areas/irregular periventricular signal extending into the deep WM; Fazekas et al., 1987). Patterns of WMHs were further assessed according to Charidimou et al. (WMH_Cha) comprising multiple (10+) subcortical spots, peri-BG WMHs, large posterior subcortical patches, and large anterior subcortical patches, each as existent or not (Charidimou et al., 2016a). Lacunes were defined as round or ovoid, fluid-filled (similar signal as CSF) cavities, 3–15 mm in diameter, visible in FLAIR images as a hypointense lumen surrounded by a hyperintense margin. They were categorized as lobar, deep, or infratentorial according to MARS. EPVSs were rated in T2-weighted images and were defined as fluid-filled hyperintense structures surrounded by small vessels with a maximum diameter of 3 mm. To increase variance, we decided to count each single EPVS. EPVSs were counted in the CSO, BG, and both hippocampi. For CSO and BG EPVS, analysis was conducted in one slice (for CSO in plane directly superior to the lateral ventricles/corpus callosum, and for BG in plane of the anterior commissure) reviewing both hemispheres, and the more severe affected side was chosen for final counting. The hippocampus was considered as a whole anatomical region, and its EPVS number is given as sum for the right and left hemisphere.

The CSVD sum score was modified from Charidimou et al. [CAA score, (Charidimou et al., 2016b)] and Staals et al. [more specific for HA (Staals et al., 2015)], which likewise take into account CMBs, cSS, WMHs, lacunes, and EPVSs. We combined both scores (Vockert et al., 2021) and extended that sum score by WMH patterns according to Charidimou et al. (2016a), hippocampal EPVSs and ICHs. The number of CMBs and EPVSs span a wide range of values, and their presence is currently predominantly rated on an ordinal scale like, e.g., the scale applied by Doubal et al. (2010) for EPVSs or Charidimou et al. for CMBs (Charidimou et al., 2016b). In detail, the CSVD sum score (CSVD-score) comprises the following variables: number of all ICHs, logarithm base 2 of number of all (whole-brain) CMBs plus one, SAH/cSS multifocality scale, FAZEKAS score (deep WMHs and periventricular WMHs separately; maximum score 6), sum of WMH patterns according to Charidimou (maximum score 4), number of all (whole-brain) lacunes, and logarithm base 2 of number of all (CSO, BG, and hippocampus) EPVS plus one. The log-transformation enabled us to differentiate between patients with medium, high, and highest count values in an unambiguous, continuous scale, while giving the CMB and EPVS components of the CSVD-score a similar weight compared to, e.g., the FAZEKAS score for WMHs. The resulting CSVD-scores were highly correlated with the results for the Charidimou (*r* = 0.93, *p* < 0.001) and Staals scores (*r* = 0.88, *p* < 0.001) extracted for our cohort, while incorporating a more diverse range of microvascular pathologies.

### Statistical Analysis

IBM SPSS Statistics 23 was used for statistical analysis. Because of the small sample size, Kruskal–Wallis tests were chosen to obtain group differences between patients with CSVD and healthy controls. Pearson's correlations were used for a comparison of continuous data, and Spearman's correlation coefficients were calculated for ordinal data. CBF, ATT, and T1eff values, which differed for more than 1.5 times the interquartile range from the median, were removed from further analysis.

## Results

### Sample

Table 1 and Supplementary Table 1 list demographical information of the study groups. Clinical diagnoses of the CSVD participants comprised cognitive decline (*n* = 3), transient ischemic attack (TIA, *n* = 2), or lacunar stroke (*n* = 2; BG and thalamus; one took place 3 years before the current MRI study), former ICHs (*n* = 2; right temporo-parietal and right parietal, respectively; both ICHs took place 4–5 years before the current MRI study) or transient focal neurological episodes (TFNEs, *n* = 1). Probable CAA was diagnosed in *n* = 4 patients, whereas *n* = 6 suffered from HA. Diagnosis of arterial hypertension was based on 24-h ambulatory blood pressure monitoring.

As expected, overall CSVD burden, e.g., the CSVD-score, was significantly higher in the CSVD group compared to controls [H(1) = 8.0, *p* = 0.005]. Global cognitive performance, assessed through the MMSE, was worse in the patient group [H(1) = 6.387, *p* = 0.011].

### Perfusion

We first compared CBF and ATT between CSVD and controls. Two outliers from the CSVD group were excluded for statistical analysis of GM, WM, and HCr CBF, and one additional CSVD outlier was excluded for BG CBF analysis (see Section Statistical Analysis for exclusion criteria). Supplementary Figure 2 shows an axial slice of the CBF result from a representative patient.

**Figure 1:**
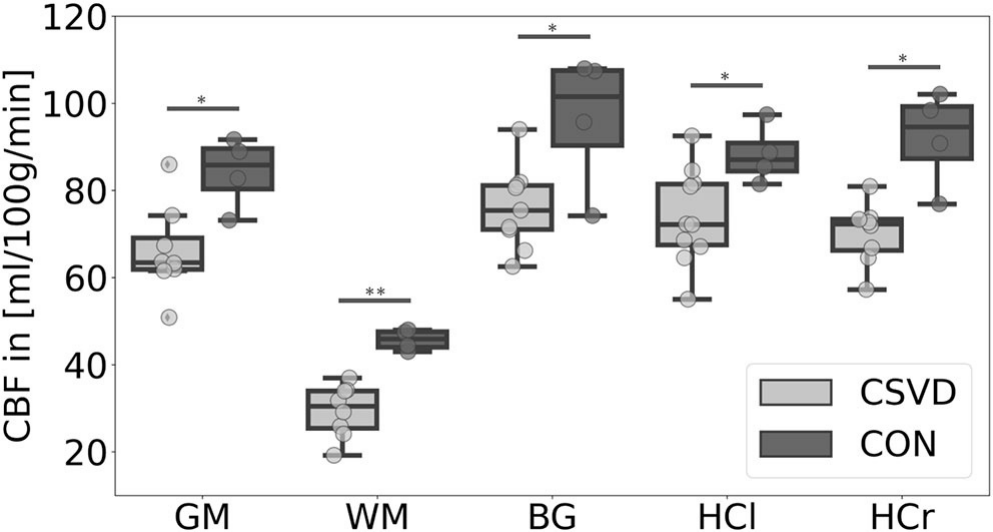
Mean cerebral blood flow (CBF) differences between patients with cerebral small vessel disease (CSVD) and controls (CON) for several regions-of-interest. Significant group differences (*p* < 0.05) of mean gray matter (GM), basal ganglia (BG), left hippocampus (HCl), and right hippocampus (HCr) CBF are marked with *; highly significant group difference (*p* < 0.01) of mean white matter (WM) CBF is marked with ^**^; outliers are not included.

**Figure 2:**
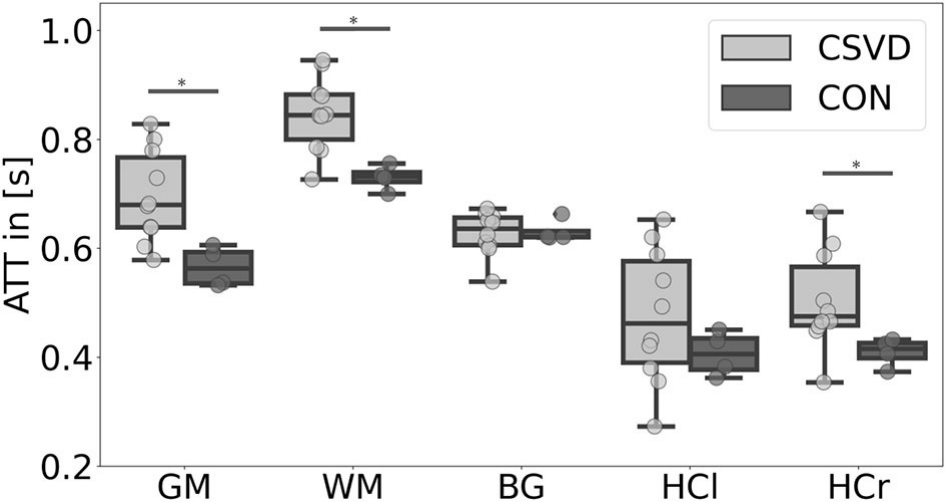
Mean arterial transit time (ATT) differences between patients with cerebral small vessel disease (CSVD) and controls (CON) for several regions-of-interest. Significant group differences (*p* < 0.05) of mean gray matter (GM), white matter (WM), and right hippocampus (HCr) ATT are marked with *; there were no significant ATT group differences for the basal ganglia (BG) and left hippocampus (HCl) ROI (*p* > 0.05).

Our data showed a significant CBF decrease in the CSVD group compared to controls affecting the GM, WM, BG, and the hippocampus (Figure 1, Table 2). This confirms previous findings of hypoperfusion in patients with CSVD, reviewed by Shi et al. (2016). Additionally, we found a significant ATT increase in patients with CSVD compared to controls in the GM, WM, and HCr ROI (Figure 2, Table 2), pointing toward a widespread impaired perfusion in patients with small vessel disease. This result correlates to findings of Dai et al. (2017), who considered the relation of increased ATT values to cerebrovascular disease.

**Table 2:** Group comparison between patients with cerebral small vessel disease and controls of the mean cerebral blood flow (CBF) and mean arterial transit time (ATT) in several regions-of-interest (ROIs).

**ROI**	**CBF**	**ATT**
Gray matter	H(1) = 4.875, *p* = 0.027	H(1) = 5.780, *p* = 0.016
White matter	H(1) = 7.385, *p* = 0.007	H(1) = 5.780, p = 0.016
Basal ganglia	H(1) = 4.024, *p* = 0.045	H(1) = 0, *p* = 1
Left Hippocampus	H(1) = 4.5, *p* = 0.034	H(1) = 0.720, *p* = 0.396
Right Hippocampus	H(1) = 6.49, *p* = 0.011	H(1) = 5.120, *p* = 0.024

*H-test results and related p-values are listed for each perfusion parameter and ROI*.

Relating perfusion results to CSVD markers, we found moderate-to-large effect size correlations between widespread hypoperfusion and greater CSVD burden (Supplementary Figure 1). This relationship was most pronounced for the WM, and there it was rather equally driven by WMHs (-Faz, -Cha), EPVSs, lacunes, and CMBs (for CBF, *r* = −0.81/−0.78/−0.70/−0.80/−0.81; for ATT, *r* = 0.66/0.60/0.68/0.67/0.77). This partially confirms findings of Rane et al. (2018) and Dolui et al. (2019), who related reduced CBF values to WMHs.

When considering lobar lacunes, lobar CMBs and CSO EPVSs separately, lacunes and CMB, but not EPVSs were driving WM/GM hypoperfusion. Conversely, for deep lacunes and CMBs, and BG EPVSs, CMBs and EPVSs were explaining BG CBF reduction (Supplementary Figure 1). As there was no consistent relationship between age, sex, and CBF or ATT (Supplementary Table 1), we did not control for these variables in our analysis.

### ASL Signal Decay Rate T1eff

Arterial spin labeling data of one participant from the CSVD group was excluded from this analysis of the signal decay rate, because of unphysiologically estimated, negative effective T1 values (probably induced by participant's motion during ASL imaging). Additionally, for the analysis of the mean signal decay rate of WM, one, and for the mean BG signal decay rate, two outliers from the CSVD group were excluded, as related values differed for more than 1.5 times the interquartile range from the median. The mean goodness (*R*^2^) of the fitting of the signal decay rate was *R*^2^ = 0.84 ± 0.19/0.86 ± 0.24/0.94 ± 0.09 for the GM/WM/BG ROI, respectively. Fit goodness was comparable between groups (*p* > 0.05).

**Figure 3:**
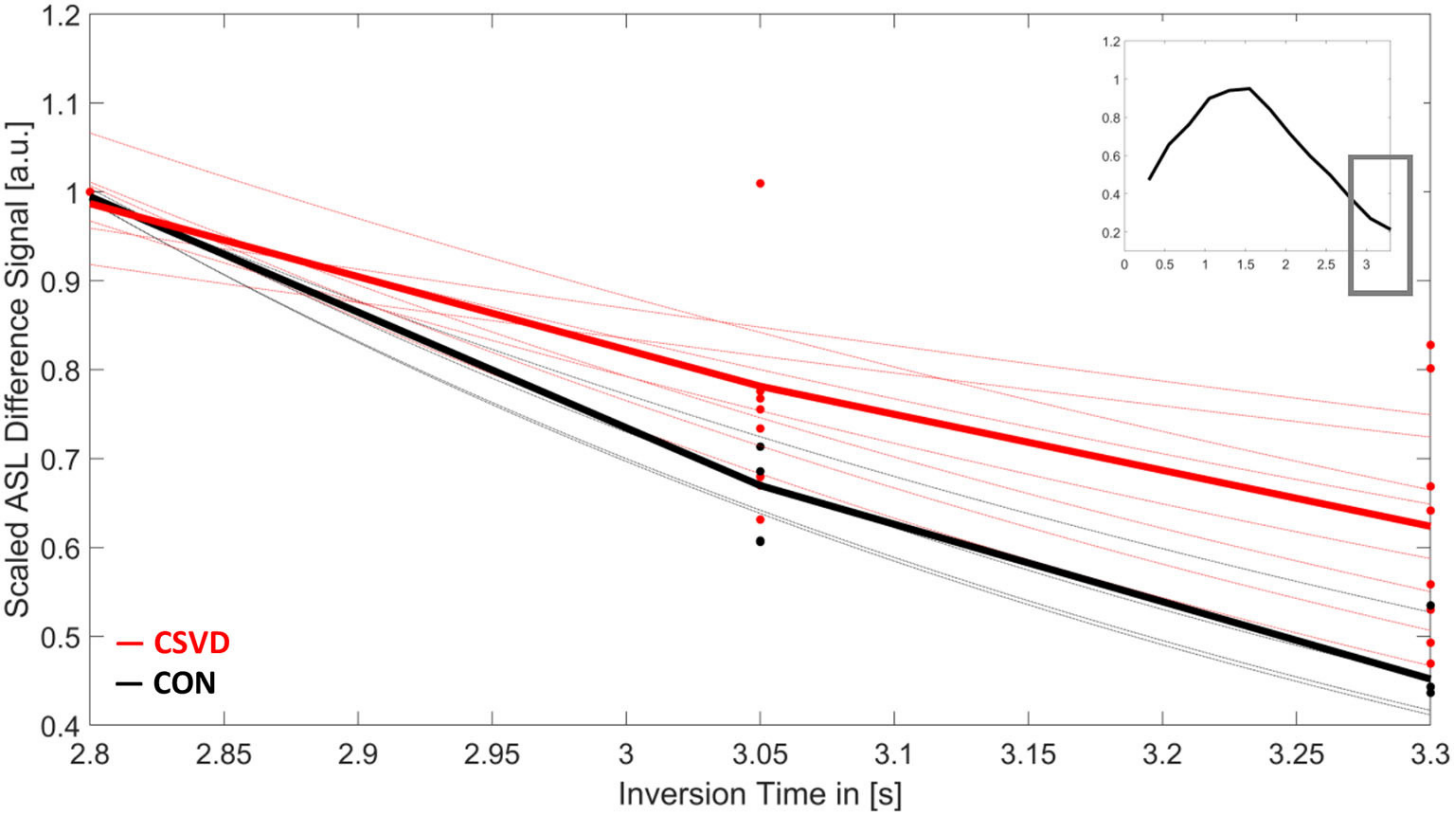
Mean arterial spin labeling (ASL) signal decay of white matter over time for patients with cerebral small vessel disease (CSVD, red) and controls (CON, black). Shown is the scaled ASL difference signal for the last three inversion times (TI). Each dot represents the measured value of each participant at TI = 2,800/3,050/3,300 ms. Thin lines show the results for the individual signal fits. Bold lines are the mean signal decays for both study groups. The small image in the upper right corner shows the mean-scaled ASL difference signal of all participants for the whole ASL time series.

**Figure 4:**
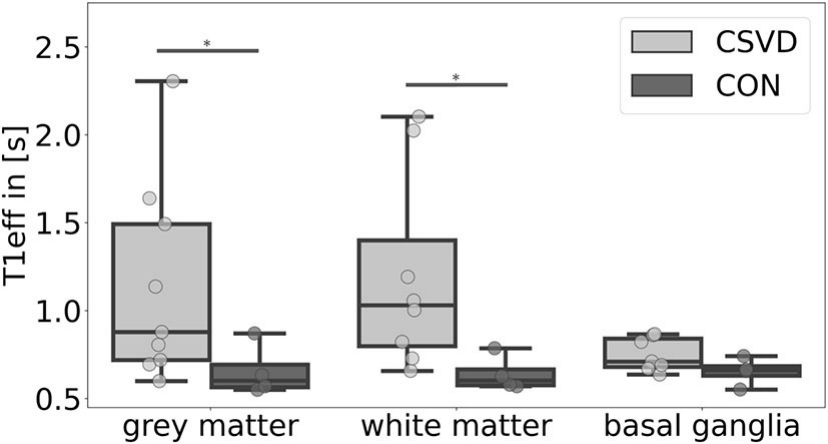
Mean effective T1-relaxation time (T1eff) differences between patients with cerebral small vessel disease (CSVD) and controls (CON) for several regions-of-interest. Significant group differences (*p* < 0.05) of mean gray matter and white matter T1eff are marked with *.

We found significant group differences of the estimated T1eff in GM [H(1) = 4.024, *p* = 0.045] and WM [H(1) = 5.654, *p* = 0.017], whereas results for the BG [H(1) = 2.286, *p* = 0.131] did not reach a level of significance (Figures 3, 4). This corresponds to results of Joseph et al. (2020), who found a decelerated signal decay in patients with AD.

Relating T1eff results to CSVD markers, we found moderate-to-large effect size correlations between greater T1eff in the WM and GM and higher CSVD burden (Supplementary Figure 1). In the WM, this association was rather equally driven by WMHs (-Faz, -Cha), EPVSs, and CMBs (*r* = 0.88/0.84/0.90/0.89), and in the GM mainly WMH-Faz and CMBs (*r* = 0.72/0.75) contributed to this relation.

When considering lobar lacunes, lobar CMBs and CSO EPVSs separately, lacunes and CMBs, but not EPVSs were driving T1eff in the WM. Additionally, there was a moderate effect size correlation between T1eff and perfusion in the WM and GM ROI (Supplementary Figure 1).

## Discussion

In this pilot study, we investigated a noninvasive ASL MRI technique to simultaneously assess brain perfusion and T1eff in patients with CSVD.

Our main findings comprise widespread hypoperfusion in the GM, WM, and subcortical regions (BG, hippocampus) in CSVD, which was related to greater small vessel disease burden, especially in the WM, where it was rather equally driven by several CSVD features. Considering EPVS region-wise, in the BG, they were related to local flow reduction, whereas CSO EPVSs did not account for GM/WM hypoperfusion. T1eff, as a potential measure of clearance, was increased in the GM and WM of patients with CSVD, but not in their BG, which was—again—related to several CSVD markers, but not necessarily to CSO EPVS burden.

Most previous studies focused on the relation of greater WMH severity and CBF decrease in CSVD (Shi et al., 2016; Rane et al., 2018; Dolui et al., 2019). However, we here found that regional hypoperfusion was also strongly related to other CSVD markers, like CMBs, EPVSs, and lacunes. A manifested CBF reduction might therefore be an indicator for total CSVD burden.

Additionally, we observed increased mean ATT in GM and WM in the CSVD group compared to controls. These longer ATTs may not only reflect micro- but also macrovascular changes (Dai et al., 2017) due to an age- and/or disease-related reduction of the alongside vessel wall stiffness (Agarwal and Carare, 2020) and associated increases of the vessel curvature. Indeed, at least stenoses/occlusions of the brain-supplying large arteries have been excluded in our cohort, which therefore did not drive the hemodynamic changes. An increase of this vessel tortuosity was found to raise severely from an age of 40 on and older (Wenn and Newman, 1990), and also typically in SVD (Agarwal and Carare, 2020). Moreover, Chen et al. found that the tortuosity of the internal carotid artery was strongly related to the number of EPVSs in the BG (Chen et al., 2020). We here could complement this finding, as in our study, the number of BG EPVSs correlated positively with ATT and also negatively with CBF. Pathological CBF reductions will change the brain's homeostasis, which leads to a degeneration of neurons and eventually to dementia (Agarwal and Carare, 2020). Future CSVD perfusion studies should therefore include microvascular arterial tortuosity measures such as a high-resolution time-of-flight angiography.

The quantification of CBF was based on the general kinetic model. The ASL difference signal of an imaged voxel is related to the amount of labeled arterial blood water delivered to this voxel by venous outflow (although usually negligible) and by T1-relaxation, whereas the latter is affected by the blood water extraction within the capillaries and the exchange with tissue water (Buxton et al., 1998). Assuming that (parts of) the labeled blood water molecules pass the blood–cerebrospinal fluid barrier and enter perivascular clearance pathways, any retardation in clearance will lead to changes in the T1-relaxation, and thus to a decelerated signal decay.

The single-compartment model assumes that there is an instantaneous exchange of labeled blood water and tissue in the capillaries and that the signal of labeled blood water therefore decays with T1 of tissue. The mean WM T1eff of the control group in this study confirmed reported values for WM T1-relaxation times at 3T (Bojorquez et al., 2017) and would therefore support this model assumption. However, due to a finite permeability of the capillary walls, labeled blood water does not exchange *immediately* with tissue. Furthermore, some labeled blood water protons may even not exchange with tissue at all, resulting in a water-extraction fraction < 1 (Parkes and Tofts, 2002). Yet, blood–brain barrier (BBB) disruptions increase the permeability of capillary walls, thus increase the water-extraction fraction, which is additionally associated with perfusion decreases (Silva et al., 1997). Cerebral capillaries are also involved in the CSF and interstitial fluid (ISF) production (Agarwal and Carare, 2020), which are essential for the brain's clearance (Xie et al., 2013). We, therefore, suggest that observed T1eff increases are related to clearance impairments. Future studies should address the question, whether the decelerated signal decay originates from a hampered extraction of water due to a reduction of the capillary density, so that more labeled blood water spins remain in the blood compartment.

For the estimation of the effective T1-relaxation, we only considered the last three TIs (TI = 2,800/3,050/3,300 ms), as a trade-off between acquisition time and an accurate perfusion estimation, to omit signal contributions of the arterial input function including bolus dispersion effects and variances of the estimated bolus arrival in the voxel. Although the T1-relaxation time of blood is shorter in men compared to women (Zhang et al., 2013), we did not incorporate any sex factors into the model of the ASL signal decay. Individual differences of the shape of the magnetically labeled blood bolus within the vessels, dependent on the labeling process and dispersion (Zhang et al., 2021), may have contributed to the total variance. Compared to Joseph et al., we fitted the ASL signal decay exponentially in respect to the Bloch equations instead of linearly. Although the T1-relaxation dominated in ASL acquisitions of this study, due to its short TE, simultaneously occurring spin–spin interactions, reflected as T2-relaxation, contributed to the overall signal decay as well. Future studies using T2-weighted ASL sequences might strengthen the hypothesis of labeled blood water spins exchanging with tissue and/or CSF.

Here, we found a relation of T1eff (perivascular clearance) and overall CSVD severity. But, there might even be regional differences of the clearance function between CAA and HA, which could be potentially resolved in future studies with larger sample sizes. In relation to the literature, could comparable studies on a larger size of patients with CAA possibly even show an association between clearance failure and CSO EPVS. Another approach of restricting T1eff analysis to the location of PVSs could be evaluated using high-resolution T2-weighted and high-resolution ASL images obtained with high-field 7T MRI.

Benefits of this ASL approach to assess impaired clearance in patients with CSVD are its noninvasiveness, the short acquisition time, the possibility to estimate yet cerebral perfusion, which might be of high interest in CSVD examinations, and the relatively simple procedure of fitting the ASL signal decay. If just clearance rates are of interest, the acquisition time could even be reduced to the later inversions times, making an application in a hospital environment for patients even more suitable.

Limitations of this pilot study are its small sample size. With a larger sample size and increased statistical power, we will be able to draw more precise and reliable conclusions, which will deepen our knowledge of microvascular impairments in patients with CSVD. In addition, the individual caffeine uptake before the MRI examinations was not controlled for, although it is well-known that caffeine alters cerebral perfusion (Mutsaerts et al., 2015) and might therefore also have an influence on clearance functions, yet T1eff. Additionally, individual hematocrit values have an impact on the T1-relaxation (Lu et al., 2004; Rosmini et al., 2019), which was not considered in this study. Furthermore, future analysis should include a potential effect of serum albumin, a substance that reduces due to inflammatory processes and is associated with increases of the vascular permeability (Fleck et al., 1985; Rozga et al., 2013). Although Rosmini et al. did not find a relation between T1-relaxation and serum albumin levels, it might have an impact to the clearance process (Rosmini et al., 2019). Further factors that contribute to the perfusion signal variance are serum glucose, blood viscosity, and the arterial pressure (Schaeffer and Iadecola, 2021). Future studies may also include the APOE4 status due to its association with BBB disruptions (Jackson et al., 2021).

This pilot study was aimed to investigate the feasibility to use a noninvasive ASL MRI approach to assess T1-relaxation times of labeled blood water, which possibly reflect clearance dysfunction in CSVD. Here, we found indications that the presented ASL method is indeed able to depict perfusion and T1eff differences in patients compared to elderly controls, and that T1eff is highly associated with perfusion and CSVD severity.

## Data Availability Statement

The raw data supporting the conclusions of this article will be made available by the authors, without undue reservation.

## Ethics Statement

The studies involving human participants were reviewed and approved by Ethics Committee of the Otto-von-Guericke University Magdeburg, Germany. The patients/participants provided their written informed consent to participate in this study.

## Author Contributions

KN analyzed the data and wrote this article. MG generated the used PASL sequence. ED supervised this work. SS performed clinical examinations (inclusive visual MRI analysis according to STRIVE) and supervised this study. All authors discussed the results and proofread this article. All authors contributed to the article and approved the submitted version.

## Funding

Funded by the Deutsche Forschungsgemeinschaft (DFG, German Research Foundation) – Project-ID 425899996, and the Federal Ministry of Education and Research (BMBF).

## Conflict of Interest

MG receives royalties from Siemens Healthineers for technology using ASL and was employed by mediri GmbH. The remaining authors declare that the research was conducted in the absence of any commercial or financial relationships that could be construed as a potential conflict of interest.

## Publisher's Note

All claims expressed in this article are solely those of the authors and do not necessarily represent those of their affiliated organizations, or those of the publisher, the editors and the reviewers. Any product that may be evaluated in this article, or claim that may be made by its manufacturer, is not guaranteed or endorsed by the publisher.

## Supplementary Material

Supplementary Figure 1 Correlation matrix of the CSVD sum score (CSVD-score), white matter hyperintensities (WMHs) related to FAZEKAS scale (WMH-Faz) and Charidimou (WMH-Cha), lacunes, lobar and deep lacunes, total number of enlarged perivascular spaces (EPVSs) in the basal ganglia, centrum semiovale, and hippocampus (EPVS), EPVSs in the centrum semiovale and basal ganglia (EPVS-CSO and EPVS-BG), microbleeds (CMBs), lobar and deep CMBs, mean cerebral blood flow (CBF), mean arterial transit time (ATT) and mean effective T1-relaxation time (T1eff) in gray matter (GM), WM, basal ganglia (BG), and for the perfusion results additionally for the left hippocampus (HCl) and right hippocampus (HCr). Shown are positive (red) and negative (blue) correlation coefficients between all parameters.

Supplementary Figure 2 Axial slice of the MPRAGE **(A)**, FLAIR **(B)**, and cerebral blood flow (CBF) result **(C)** of a representative patient (ID = 5). Estimated CBF results (in structural space) before a partial volume correction was applied are shown, to visualize gray and white matter values within the same image.

Supplementary Table 1 Demographical information and individual study results used for statistical analysis.
