# Supplementary figures and images for: Microvascular Impairment in Patients With Cerebral Small Vessel Disease Assessed With Arterial Spin Labeling Magnetic Resonance Imaging: A Pilot Study
Source: Front Aging Neurosci. 2022 May 19;14:871612. doi: 10.3389/fnagi.2022.871612 (PMC9161030; doi:10.3389/fnagi.2022.871612)

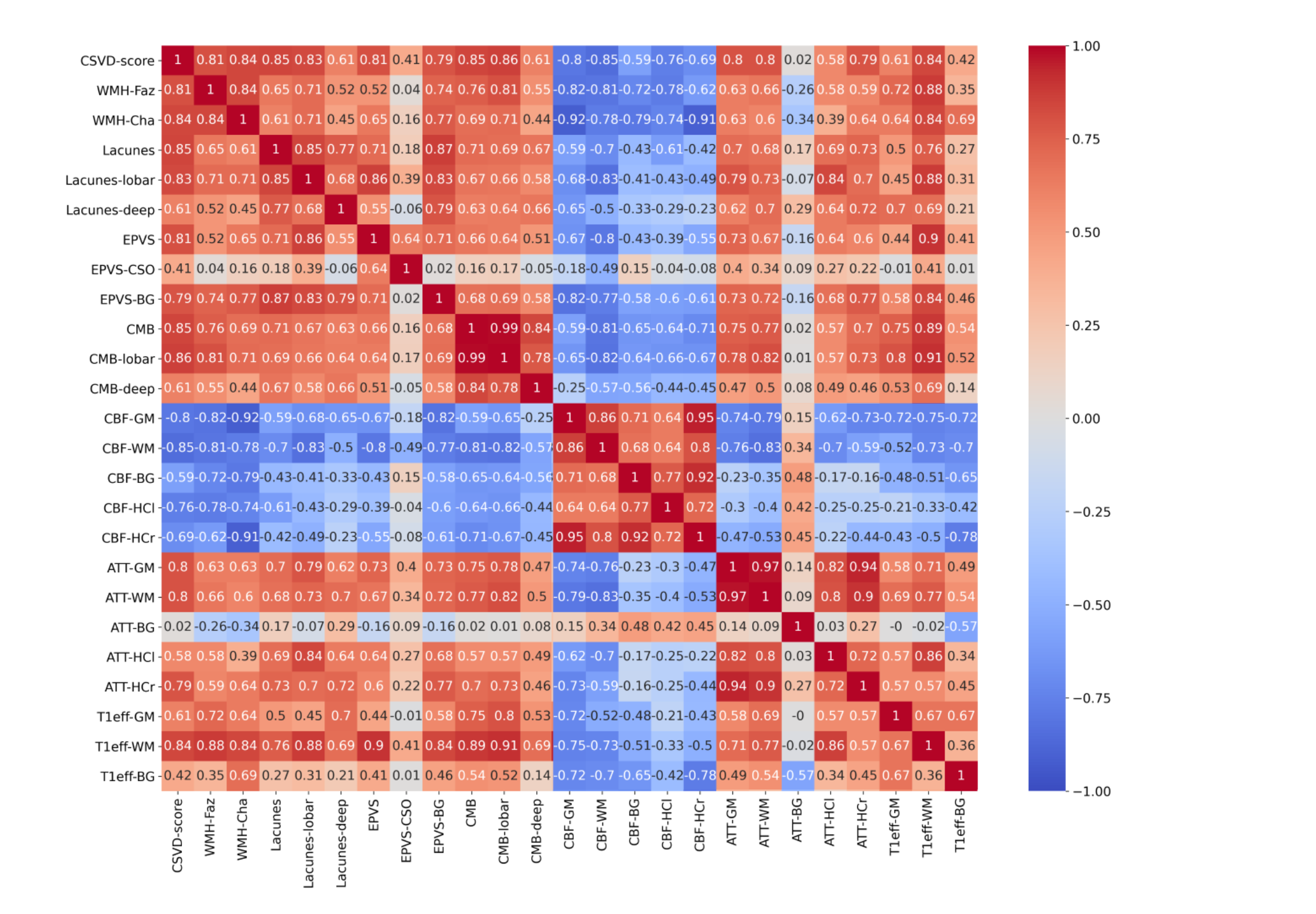

Supplement: Supplementary Figure 1 — Correlation matrix of the CSVD sum score (CSVD-score), white matter hyperintensities (WMHs) related to FAZEKAS scale (WMH-Faz) and Charidimou (WMH-Cha), lacunes, lobar and deep lacunes, total number of enlarged perivascular spaces (EPVSs) in the basal ganglia, centrum semiovale, and hippocampus (EPVS), EPVSs in the centrum semiovale and basal ganglia (EPVS-CSO and EPVS-BG), microbleeds (CMBs), lobar and deep CMBs, mean cerebral blood flow (CBF), mean arterial transit time (ATT) and mean effective T1-relaxation time (T1eff) in gray matter (GM), WM, basal ganglia (BG), and for the perfusion results additionally for the left hippocampus (HCl) and right hippocampus (HCr). Shown are positive (red) and negative (blue) correlation coefficients between all parameters. [file Figure_1.TIF]

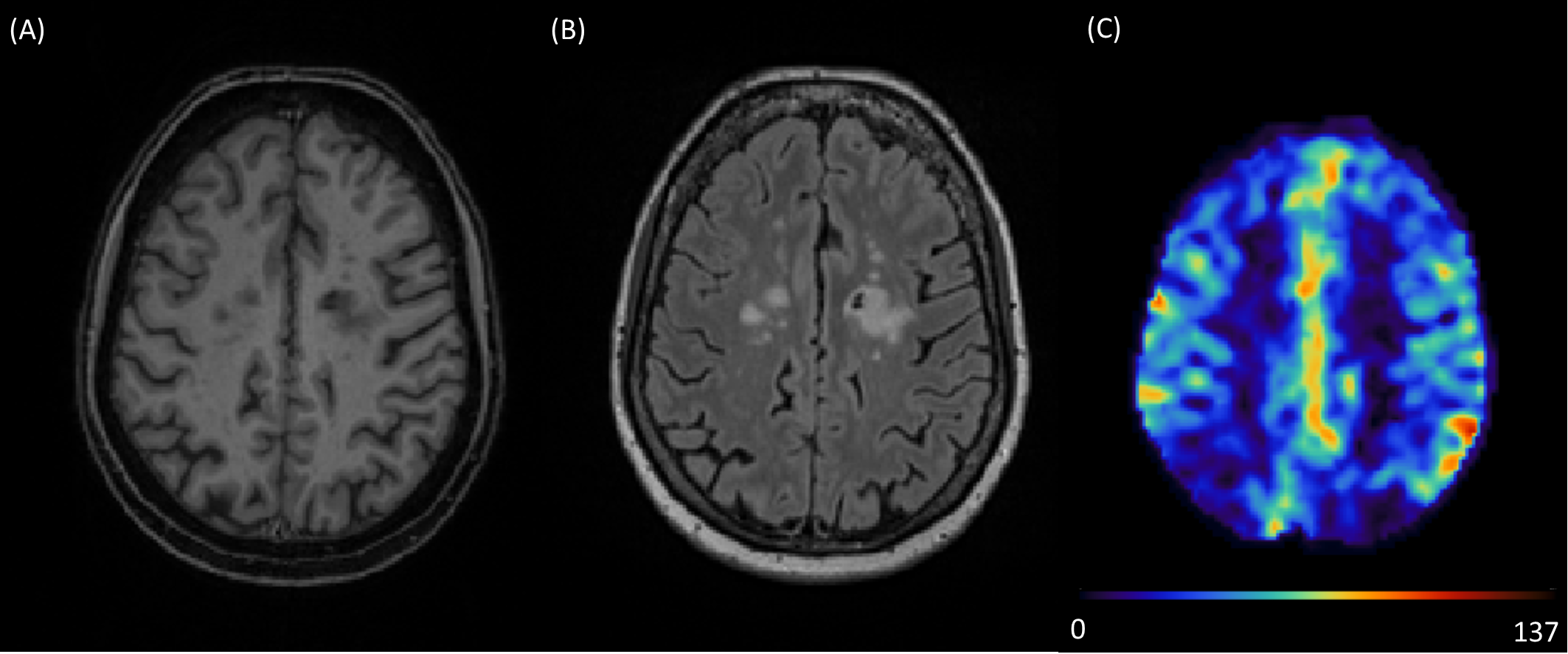

Supplement: Supplementary Figure 2 — Axial slice of the MPRAGE (A), FLAIR (B), and cerebral blood flow (CBF) result (C) of a representative patient (ID = 5). Estimated CBF results (in structural space) before a partial volume correction was applied are shown, to visualize gray and white matter values within the same image. [file Figure_2.TIF]
